# Long-Term Outcomes of Uterine Artery Embolization for Treatment of Fibroids in Women Under 40: A Retrospective Survey Study at Two Institutions with Median 16-year Follow-up

**DOI:** 10.1007/s00270-026-04402-w

**Published:** 2026-03-27

**Authors:** Elisabeth R. Seyferth, Neil E. Harrison, Richard D. Shlansky-Goldberg

**Affiliations:** Division of Interventional Radiology, Department of Radiology, University of Pennsylvania Perelman School of Medicine, Philadelphia, PA USA

**Keywords:** Uterine fibroid embolization, Uterine artery embolization, Fibroid, Leiomyoma, Dysfunctional uterine bleeding, Menorrhagia

## Abstract

**Purpose:**

To assess long-term freedom from symptom recurrence and success of entering menopause without hysterectomy for patients younger than 40 years old undergoing uterine fibroid embolization (UFE).

**Materials and Methods:**

Women under 40 who underwent UFE from 2001–2012 at two institutions and who participated in a telephone survey in 2024 were included. The survey assessed symptom response to UFE, need for additional procedures, and patient satisfaction with UFE.

**Results:**

976 women underwent UFE; of these, 173 were younger than 40 at the time of UFE, and 80 of 173 (46.2%) participated in the survey. Median age at embolization was 37.2 (range 26.4–39.8) and when surveyed was 52.7 (range 43.1–61.9). 70.0% (56/80) and 18.8% (15/80) of patients reported “a lot” or “a little” symptom improvement, respectively, in the first 6 months after UFE; 11.2% (9/80) reported no change. Of those with improvement, symptoms returned in 57.7% (41/71) with median time to recurrence of 11.0 years (range 0.5–20 years). Freedom from recurrence of symptoms was 68/71 (95.8%) at 1 year, 48/71 (67.6%) at 5 years, and 36/71 (50.7%) at 10 years. Freedom from hysterectomy for recurrent symptoms was 70/71 (98.6%) at 1 year, 60/71 (84.5%) at 5 years, and 51/71 (71.8%) at 10 years. Patient satisfaction with having UFE was a median of 5 on a scale of 1–5 (5 = very satisfied).

**Conclusion:**

Women under 40 who undergo UFE are likely to experience return of symptoms before menopause and may require additional procedures, yet they remain highly satisfied with the procedure.

**Level of Evidence:**

Level 4, Case Series

**Graphical Abstract:**

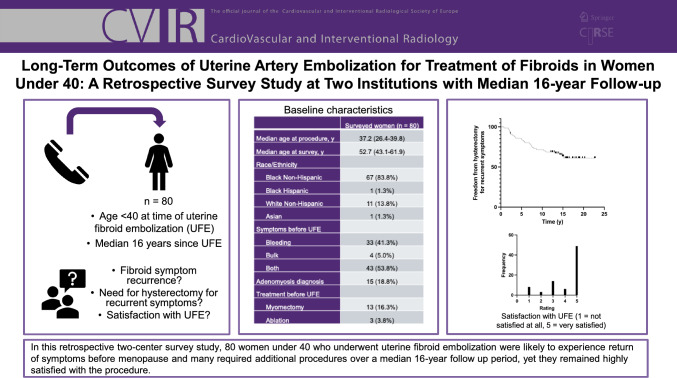

**Supplementary Information:**

The online version contains supplementary material available at 10.1007/s00270-026-04402-w.

## Introduction

Uterine fibroids are found in up to 70% of women before menopause, and up to 25% of women have fibroid-related symptoms requiring intervention [1–3]. Uterine fibroid embolization is a minimally-invasive treatment for fibroid-related symptoms that works by causing fibroid infarction [4, 5]. Multiple randomized controlled studies have shown comparable clinical efficacy, fibroid-related quality of life scores, and major adverse event rates between UFE and myomectomy, although UFE is associated with a shorter hospital stay and faster recovery [6–8]. Randomized trials and meta-analyses of UFE versus focused ultrasound have found a lower reintervention rate and greater symptom improvement in the UFE group [9, 10].

Less is known about outcomes in patients who undergo UFE at younger ages. One retrospective study found a significantly higher likelihood of treatment failure in UFE patients under 40 compared to those older than 45, and one randomized trial found that younger patient age was an independent predictor of reintervention following initial treatment, although few patients under 40 were included [9, 11]. In addition, there are few studies assessing outcomes following UFE with more than 5 years of follow up, particularly in younger patients, but studies of other uterine-sparing fibroid treatments have found recurrence rates at 5 years as high as 52.9% [12]. These patients may be more likely to require additional procedures for recurrent symptoms given the number of years remaining before their mean expected onset of natural menopause at age 51 [13]. Additionally, patients who develop symptomatic fibroids at younger ages may be more likely to continue to develop fibroids after initial treatment, as genetic, hormonal, and environmental factors affecting individuals have been implicated in fibroid growth [14]. The goal of this study was to assess symptom recurrence, success of entering menopause without hysterectomy, and long-term satisfaction in patients under the age of 40 who underwent UFE.

## Materials and Methods

### Data Collection

Institutional review board approval and a waiver of informed consent was obtained for this retrospective study. All women who underwent UFE at two institutions from 2001 to 2012 were identified; this study period was chosen to include women who had UFE in their 30s who are now likely to be postmenopausal. Patients were identified using either a quality assurance database (HI-IQ®, ConexSys, Lincoln, Rhode Island) or a database of imaging reports (Nuance® mPower™ “Montage,” Nuance Communications, Inc., Burlington, Massachusetts). Women who were under 40 at the time of embolization were identified and called between 8/29/24 and 10/3/24 by a single interventional radiology resident with prior experience in survey administration. Those who answered were asked if they would be willing to take an 11-question survey administered over the phone (Supplemental Information, Appendix A). Women were excluded if they did not answer the phone after a minimum of 3 call attempts on different dates, had a wrong or disconnected number, or refused to participate. Patients who reported some improvement in symptoms followed by recurrence within 3 months were designated as having no improvement given lack of sustained response over even a short follow-up period.

Additional baseline patient information was gathered from the electronic medical record (EMR), and survey responses were correlated to information from the EMR when available. If there was a discrepancy between a response and the EMR (for example, the date of a hysterectomy), the information from the EMR was imputed for data analysis. If only the year that a hysterectomy was performed was known, it was imputed as being performed on July 1 of that year. For patients who had a hysterectomy following embolization, the operative and pathology results were reviewed, if available, to evaluate for additional diagnoses that may contribute to symptoms.

### Outcomes and Definitions

The primary outcome as assessed by phone survey (Supplemental Information, Appendix A) was freedom from hysterectomy for recurrent symptoms prior to menopause in women who underwent UFE in their 30s. Secondary outcomes were patient satisfaction and success of pregnancy, defined as successful live birth, if attempted after UFE. The survey assessed initial treatment response (assessed in the survey as “a lot of improvement,” “a little improvement,” “no improvement,” or symptom “worsening” in the first 6 months after UFE), long-term treatment response (assessed by asking patients whose symptoms initially improved whether their symptoms recurred or worsened again), need for additional procedures to treat these symptoms (including hysterectomy), and patient satisfaction with UFE. Perimenopausal status was defined as having intermittent menses and possible associated symptoms such as hot flashes, night sweats, or sleep disturbance. Menopausal status was defined as having cessation of menses for at least one year.

### Techniques

UFE was performed under moderate sedation. Bilateral uterine artery embolization was performed in all cases. Tris-acryl gelatin microspheres (Embosphere® Microspheres, Merit Medical Systems Inc., South Jordan, Utah) were used in 71.3% of procedures and polyvinyl alcohol (PVA) particles (Contour™, Boston Scientific, Marlborough, Massachusetts) in 28.7%. Embolization was initiated with microspheres or particles in the 300–500 μm size range in 21.3% of cases, 500–700 μm in 75.0%, and 700–900 μm in 1.3% (particle size was unknown in 2 procedures). Embolization endpoint was a “pruned tree” appearance or vascular stasis. All procedures in this study were technically successful.

### Statistical Analysis

Summary statistics were reported as median and range for continuous data and as count with percentages for categorical data. Chi square test was used to evaluate likelihood of symptom recurrence between age groups. Binomial logistic regression was used to assess whether patient age was correlated to symptom recurrence and need for hysterectomy in patients who initially responded to UFE. Kaplan-Meier analyses were used to evaluate the time to recurrence of fibroid-related symptoms for patients with initial improvement and time to additional treatment for recurrence. Cox proportional hazards regression was used to assess whether patient age was correlated to time to symptom recurrence and time to hysterectomy in patients who initially responded to UFE. Statistical analyses were performed using GraphPad Prism Version 10.3.1 (GraphPad Software, Boston, Massachusetts).

## Results

### Study Population

**Fig. 1:**
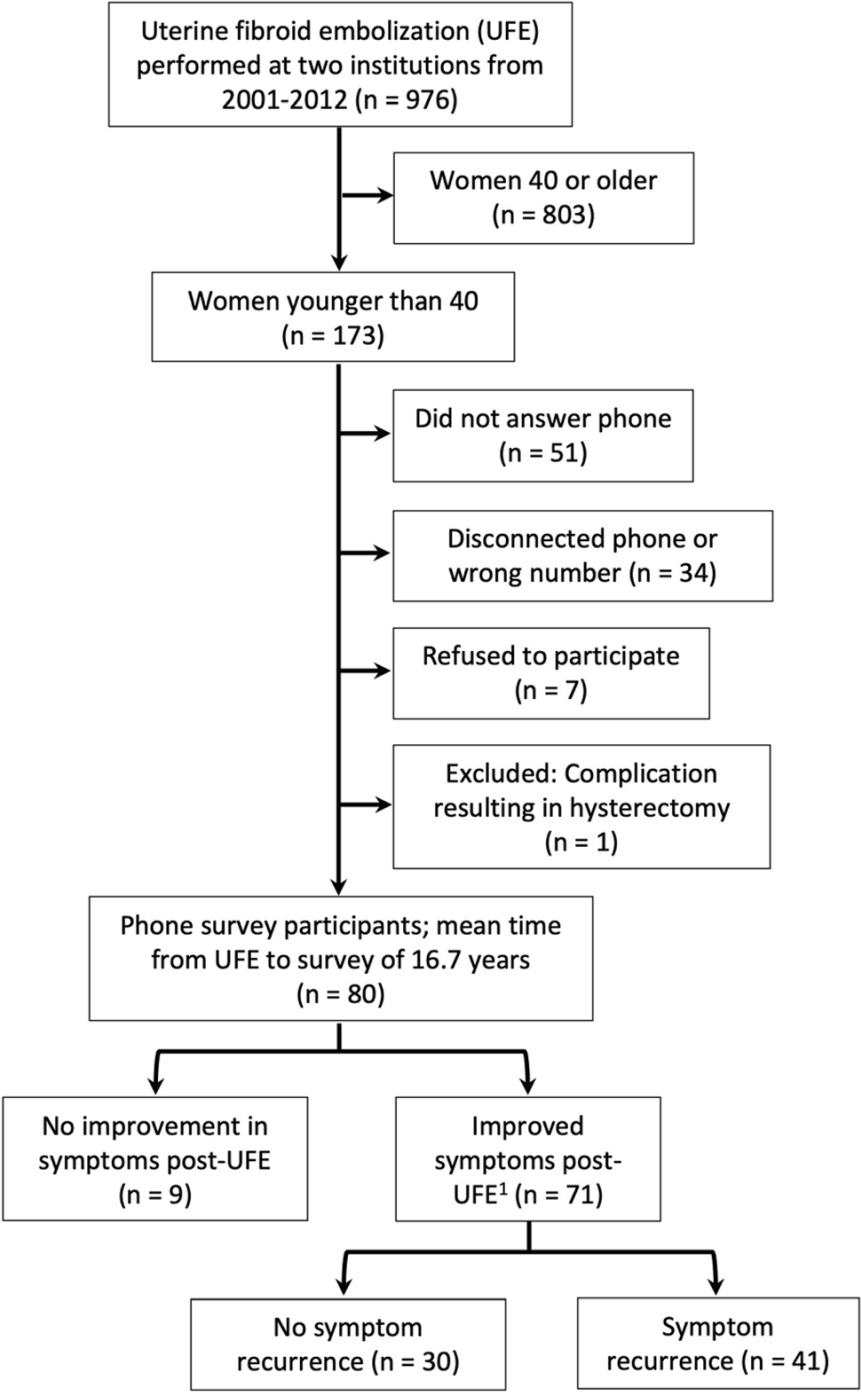
Flowchart of the study population. ^1^Includes patients who reported “a lot” or “a little” improvement in fibroid symptoms after UFE

976 women who underwent UFE at two institutions from 2001 to 2012 were identified (Fig. 1). 173 women who were under 40 at the time of embolization were identified and called. 81 women completed the phone survey. 51 women did not answer the phone after a minimum of 3 call attempts on different dates, 34 had a wrong or disconnected number, and 7 refused to participate. One patient was excluded from analysis of symptom outcomes as she reported having a hysterectomy soon after the procedure due to a complication.

Baseline characteristics of the 80 patients included in the study are shown in Table 1. Long-term outcomes or phone survey data from the included cohort have not been previously published. Median age at time of embolization was 37.2 years (range 26.4–39.8), median age at time of survey was 52.7 years (range 43.1–61.9), and median time from embolization to survey was 16.1 years (range 11.9–22.8). 56 embolizations were performed at one institution and 24 at the other. 18.8% of women (15/80) had a combination of fibroids and adenomyosis by EMR or self-report. 41.3% of patients (33/80) reported bleeding symptoms only, 5.0% (4/80) bulk symptoms only, and 53.8% (43/80) both bleeding and bulk symptoms. 16.3% of patients (13/80) had prior myomectomy and 3.8% (3/80) had prior endometrial ablation.

**Table 1:** Baseline characteristics of 80 surveyed patients

	Count or median
Median age at procedure, y	37.2 (26.4–39.8)
Median age at survey, y	52.7 (43.1–61.9)
Hospital 1	56 (70.0%)
Hospital 2	24 (30.0%)
Race/ethnicity	
Black non-hispanic	67 (83.8%)
Black hispanic	1 (1.3%)
White non-hispanic	11 (13.8%)
Asian	1 (1.3%)
Symptoms before UFE	
Bleeding	33 (41.3%)
Bulk	4 (5.0%)
Both	43 (53.8%)
Adenomyosis diagnosis	15 (18.8%)
Treatment before UFE	
Myomectomy	13 (16.3%)
Ablation	3 (3.8%)

### Treatment Response and Recurrence

**Fig. 2:**
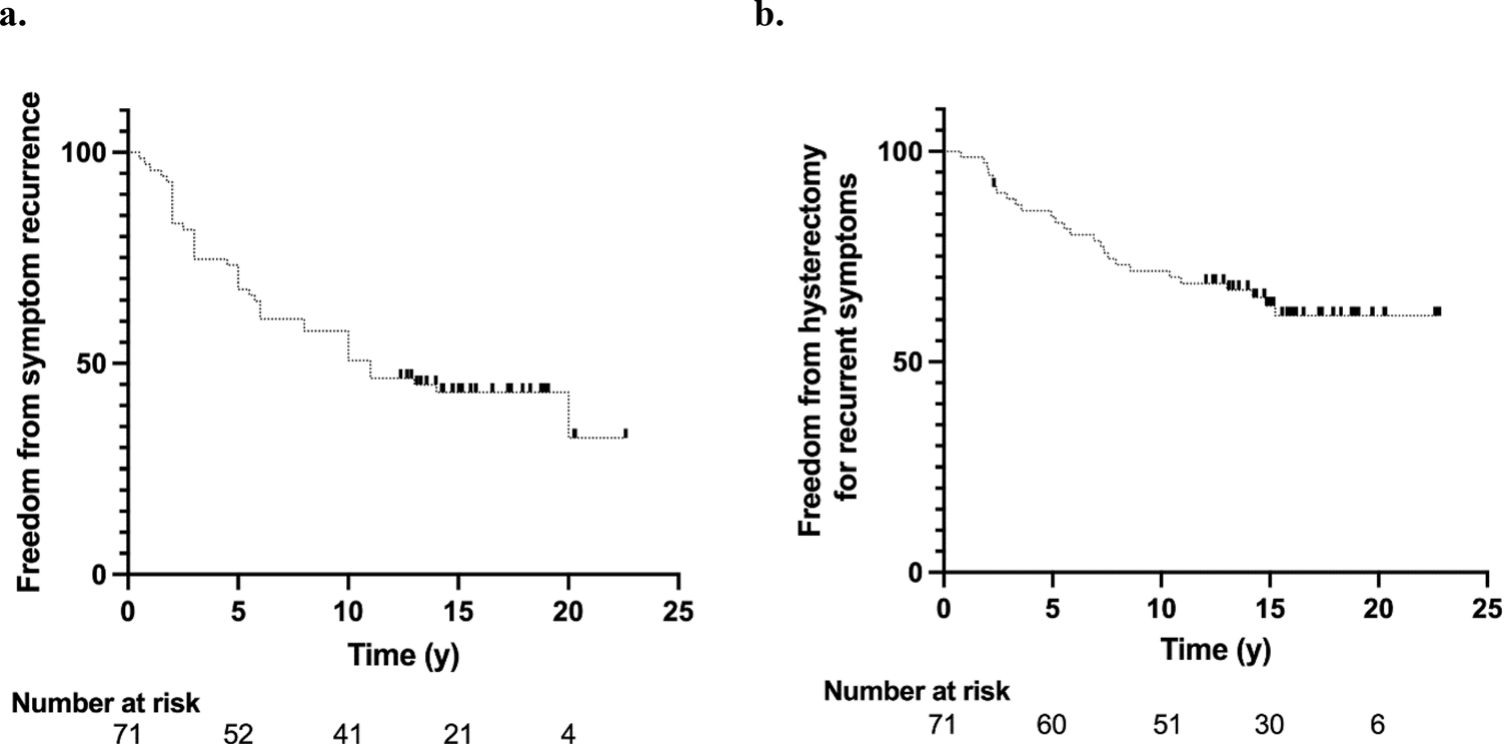
Kaplan-Meier curves of **a** freedom from recurrence or worsening of fibroid-related symptoms in all patients with “a lot” or “a little” initial improvement after UFE and **b** freedom from hysterectomy among all patients with initial symptomatic improvement following UFE

70.0% of women (56/80) reported that their fibroid symptoms improved “a lot” and 18.8% (15/80) “a little” in the first 6 months after UFE, while 11.2% (9/80) experienced no change (Table 2). Among the 71 patients who experienced initial improvement in symptoms, 30 (42.3%) reported that their symptoms never recurred and 41 (57.7%) reported that their symptoms returned or worsened. Median time to symptom recurrence or worsening was 11.0 years (range 0.5–20 years; Fig. 2A; Supplemental Information, Appendix B). Freedom from recurrence or worsening of fibroid-related symptoms was 68/71 (95.8%) at 1 year; 48/71 (67.6%) at 5 years; and 36/71 (50.7%) at 10 years. When grouped into thirds by age, the youngest patients were significantly more likely to experience recurrence (19/24, 79.2%; versus 12/24, 50.0% likelihood in middle third and 10/23, 43.5% likelihood in oldest third; *p* = 0.03 across the three groups) (Table 3). When age was included as a continuous variable in a logistic regression model, there was a trend towards greater likelihood of symptom recurrence in younger patients (odds ratio 0.85, 95% confidence interval 0.70–1.00, *p* = 0.07). Kaplan-Meier analysis found no association of patient age at time of UFE with time to symptom recurrence.

**Table 2:** Uterine fibroid embolization outcomes in 80 surveyed patients

	Count or median
Treatment response in first 6 months	
A lot better	56 (70.0%)
A little better	15 (18.8%)
No change	9 (11.2%)
No recurrence in those with initial improvement	30 (42.3%)
Symptom recurrence in those with initial improvement	41 (57.7%)
Underwent procedure(s) after UFE for recurrence	30 (37.5%)
Post-UFE treatments for recurrence	
Repeat UFE	3 (3.8%)
Myomectomy	3 (3.8%)
Endometrial ablation	1 (1.3%)
Hysterectomy for fibroids	26 (32.5%)
Attempted pregnancy	10 (12.5%)
Successful pregnancy in those who attempted	4 (40.0%)
Menopause status, of those with no hysterectomy (n = 49)	
Premenopausal	8 (16.3%)
Perimenopausal	13 (26.5%)
Menopausal	28 (57.1%)
Median satisfaction with having UFE (scale 1–5, 5 highest)	5.0 (1–5)

**Table 3:** Likelihood of experiencing symptom recurrence among the 71 women who initially responded to uterine fibroid embolization, grouped into thirds by age

Group	N	Mean age	Experienced recurrence	*P*-value*
Youngest	24	32.9	19 of 24 (79.2%)	
Middle	24	37.4	12 of 24 (50.0%)	0.03
Oldest	23	39.2	10 of 23 (43.5%)	

^*^P-value from the X^2^ test of difference in recurrence rates across the three age groups

37.5% (30/80) of patients underwent additional procedures or surgeries to treat recurrent fibroid symptoms (Table 2) and 10.0% (8/80) underwent additional procedures after having no symptom improvement following UFE. Freedom from reinterventions for recurrent symptoms among patients who initially responded to UFE were 70/71 (98.6%) at 1 year; 59/71 (83.1%) at 5 years; and 48/71 (67.6%) at 10 years. Among the 71 patients whose symptoms initially improved after UFE, overall freedom from hysterectomy for recurrent fibroid symptoms was 63.4% (45/71; Fig. 2B). Freedom from hysterectomy for recurrent symptoms was 70/71 (98.6%) at 1 year; 60/71 (84.5%) at 5 years, and 51/71 (71.8%) at 10 years. There was no association of patient age at time of embolization with freedom from hysterectomy or time to hysterectomy for recurrent symptoms. A single patient had a hysterectomy for placenta previa causing hemorrhage after childbirth—not for fibroids—and was excluded from further analysis.

Of patients who did not have a hysterectomy, 16.3% (8/49) are currently premenopausal, 26.5% (13/49) are perimenopausal, and 57.1% (28/49) have entered menopause. Overall, 90.0% (72/80) of all included patients have either had a hysterectomy or are peri- or postmenopausal.

### Hysterectomy Pathology and Intraoperative Findings

A surgical pathology report was available for 19 of 30 patients (63.3%) who had a hysterectomy for fibroid symptoms. In addition to uterine fibroids, adenomyosis was reported in 7 of 19 (36.8%), endometriosis in 3 (15.8%), and endometrial polyps in 1 (5.3%). There were 2 patients (10.5%) for which the pathology report noted only 1–2 intramural or subserosal fibroids measuring up to around 2 cm, in whom fibroids may have been unlikely to account for their symptoms. In the remainder, pathology reports noted “numerous” or “multiple” leiomyomata of larger sizes. Mean uterine mass was 694.9 g (range 135.2–2548.4 g).

### Pregnancy Outcomes

12.5% of women (10/80) reported attempting to become pregnant after UFE. Of these, 40.0% (4 of 10) had a successful live birth. Among the 6 patients who attempted to conceive but did not have a child, one reported having a pregnancy that ended in miscarriage, one reported having an ectopic pregnancy, two reported being unable to conceive (in one case despite the use of in-vitro fertilization), and two declined to provide further details.

### Satisfaction with UFE

**Fig. 3:**
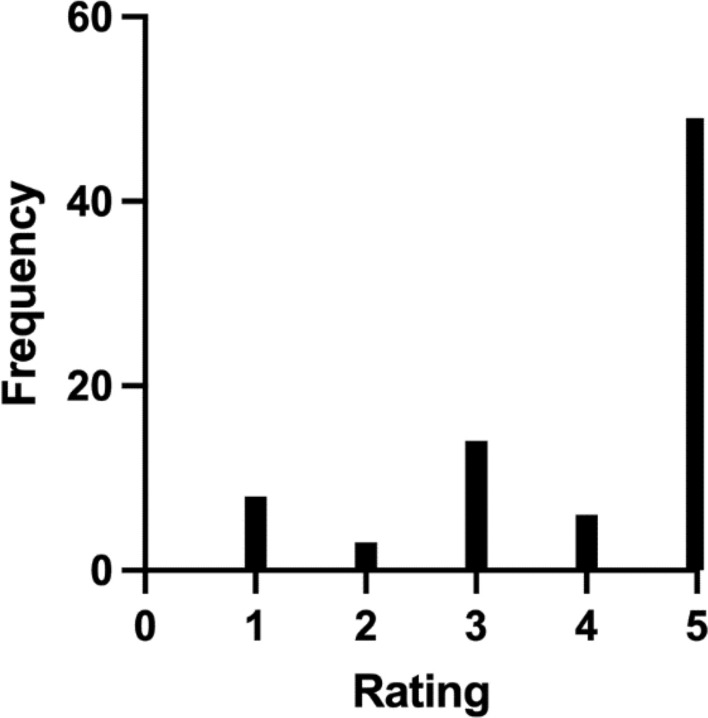
Satisfaction with uterine fibroid embolization assessed at least 12 years after embolization in patients less than 40 years old at the time of embolization (1 = not satisfied at all, 5 = very satisfied)

Median satisfaction with having UFE was 5 on a scale of 1–5, with 1 being not at all satisfied and 5 being very satisfied (Fig. 3). Eight patients rated their satisfaction with having UFE as a 1, 3 ranked it a 2, 14 ranked it a 3, 6 ranked it a 4, and 49 ranked it a 5. Common themes in patient responses when asked why a satisfaction score was chosen are shown in Table 4.

**Table 4:** Common themes of responses from 80 patients when asked why they selected their satisfaction score with having uterine fibroid embolization

	Count
Improved quality of life and/or symptoms; recommends to others	61 (76.3%)
Less invasive treatment; was not ready for hysterectomy	19 (23.8%)
Symptoms recurred	19 (23.8%)
No change in quality of life or symptoms; did not work well	8 (10.0%)
Painful procedure; severe or unmanageable pain	6 (7.5%)
Potential impact on pregnancy outcomes	4 (5.0%)

## Discussion

This multicenter study finds that UFE initially improved symptoms in nearly 90% of women younger than 40 but that, as has been seen for other uterine-sparing fibroid treatments over long-term follow-up periods, symptoms recurred in many over a median follow-up time of 16 years. Almost all patients reached by this study either have had a hysterectomy since UFE or are currently peri- or postmenopausal, allowing evaluation of long-term outcomes in a cohort of patients who underwent UFE at a young age. Comparison to other uterine-sparing treatments is limited by lack of data with such long-term follow-up in patients under 40, as well as differences in how recurrence is defined. However, Radosa et al. reported a 31.3% recurrence rate of symptomatic fibroids in patients aged 30–39 at 5 years after laparoscopic myomectomy, and Li et al. reported a 30.8% reintervention rate 10 years after treatment of patients under 40 with ultrasound-guided high intensity focused ultrasound ablation [15, 16]. Recurrence rates after laparoscopic myomectomy have ranged from 16.7 to 52.9% at 5 years in patients of all ages [12, 17]. In the current study, recurrent symptoms were more likely in younger women. This finding corroborates multiple other studies of UFE and myomectomy that have shown that younger age is an independent risk factor for recurrent fibroid symptoms after treatment [9, 11, 15, 18].

Most patients endorsed high satisfaction with UFE despite high rates of symptom recurrence. Multiple patients who chose “very satisfied” noted that UFE “changed their life,” as they were planning their life around their menstrual cycles before the procedure. This corroborates prior studies of showing high quality of life and satisfaction scores following UFE with shorter follow up periods. For example, the FEMME trial reported a mean health-related quality of life domain score on the Uterine Fibroid Symptom and Quality of Life (UFS-QOL) questionnaire of 80.0 ± 22.0 at 2 years (compared to a mean score of 42.1 ± 26.4 at baseline) post-UFE, the REST trial reported 90% of patients would recommend the treatment to a friend at 5 years, and the EMMY trial reported 78% of patients were satisfied with UFE at 10 years [6, 19, 20]. Additionally, hysterectomy in younger women is associated with a variety of negative health effects, including increased risk of all-cause mortality, stroke, coronary artery disease, and hypertension, and may have other negative long-term effects such as pelvic prolapse, incontinence, or regret over loss of fertility [21, 22]. Many patients who did not have a sustained response to embolization reported happiness with getting years of relief from the procedure and still rated it highly; others reported that they did not feel ready to have a hysterectomy at a young age and that UFE gave them time to consider it.

Notably, while some patients mentioned that they were counseled that symptoms may recur, others felt that they were not told of that possibility and therefore chose a lower score. Additionally, several patients reported giving a lower score because of possible negative impact on pregnancy outcomes or because they were told by providers after the procedure that they could not or should not try to conceive (this type of counseling is not part of our practice). Appropriate pre-procedure counseling from interventional radiology and other specialties may improve patient satisfaction and set expectations for possible outcomes. Patients who do not experience initial improvement in symptoms, whether due to technical or anatomic reasons, or who have late recurrence of symptoms, should also be counseled that UFE can be repeated if needed, potentially allowing patients to avoid hysterectomy even in these cases.

Most studies of long-term outcomes following UFE have a mean patient age in the early to mid 40s, with few focusing on long-term follow-up of patients under 40 [20, 23–26]. These studies not focused on younger cohorts have found generally lower rates of reintervention following UFE than the current study. While the reintervention rate after UAE was 32% at 5 years in the REST trial and 24% at 4.5 years in the FEMME trial, one meta-analysis found a reintervention risk after 5 years of 14.4% (95% CI 9.8%–19.6%) [10, 19, 27]. In a study with median follow up time of 5.7 years, Scheurig-Muenkler et al. found a treatment failure rate of 32% in a sub-analysis of patients under 40, with reintervention performed at a median of 1.3 years following UFE [11]. To our knowledge, no study of patients under 40 has approached the median 16.1-year follow-up of the current study, which may account for the higher rates of symptom recurrence and invasive retreatment in this cohort.

Four of 10 women who attempted to become pregnant after UFE in this study were able to have a child. While data on fertility rates after UFE from randomized trials remain limited, this finding is comparable to the reported labor rate of 48% among patients who attempted to become pregnant after myomectomy in a prior trial [28]. Recent meta-analyses have reported no difference in pregnancy outcomes between UFE and myomectomy [29, 30].

This study is limited by its retrospective nature and by relying heavily on self-report, as information from the EMR is sparse from much of the period covered. Therefore, it is subject to both recall and response bias. It is also based on a phone survey; while the response rate of 47% is within the range of many published phone survey studies, bias is imposed by patients not answering, having disconnected numbers, or refusing to participate. Additionally, a validated uterine fibroid symptoms-related questionnaire such as the UFS-QOL or other detailed quality of life questions were not used, both because the authors suspected that answers to a detailed symptom survey would not be accurate when recalling symptoms from years prior and to avoid burdening survey participants with a lengthy questionnaire [31]. This study did not include data on fibroid number, size, or location, or uterus size based on imaging prior to or after UFE due to the limited records available from EMR at the time of intervention for many patients. Additionally, 3.8% (3/80) of the patients in the study were treated with 500–700 micron spherical PVA particles, which have been associated with higher reintervention rates [32]. However, all three patients had “a lot” of improvement initially and all required no treatment for recurrent symptoms in our study. 28.7% of the patients were treated with PVA particles of any kind. While this differs from our modern technique of using tris-acryl gelatin microspheres, a previous randomized controlled trial did not show a difference in fibroid infarction outcomes with PVA versus microspheres [33]. Finally, the majority black, non-Hispanic population in this study may limit the ability to generalize its results.

## Conclusion

In conclusion, many women under 40 treated by UFE developed recurrent symptoms and required further procedures, similar to outcomes of other uterine-sparing treatments in younger women. Nonetheless, satisfaction with having undergone embolization remained high, with many women emphasizing that they did not wish to undergo hysterectomy at a young age and appreciating the years of symptom relief provided by UFE.

## Supplementary Information

Below is the link to the electronic supplementary material.

Supplementary file1 (DOCX 16 KB)

Supplementary file2 (DOCX 21 KB)
