# Supplementary material for: Long-Term Outcomes of Uterine Artery Embolization for Treatment of Fibroids in Women Under 40: A Retrospective Survey Study at Two Institutions with Median 16-year Follow-up
Source: Cardiovasc Intervent Radiol. 2026 Mar 27;49(7):1391–9. doi: 10.1007/s00270-026-04402-w (PMC13337861; doi:10.1007/s00270-026-04402-w)
Supplement: Supplementary file 1 — Supplementary file1 (DOCX 16 KB) [file 270_2026_4402_MOESM1_ESM.docx]

Appendix A. Uterine Artery Embolization Questionnaire

1. Did you have a uterine artery embolization (UAE) to treat symptoms of fibroids? *(If patient answers “no,” stop questionnaire.)*
2. What symptoms did you have that led you to have a UAE performed? *(specifically ask patient about bulk symptoms (bloating, frequent urination, pain with sex) if they only endorse bleeding symptoms, and vice versa).*
3. Did you have any other procedure or surgery done to treat fibroids before the UAE? *(endometrial ablation, myomectomy)*
   1. If yes, please list the other procedure(s).
4. Have you been diagnosed with adenomyosis as well?
5. How did your fibroid symptoms change in the first 6 months after the UAE?
   1. A lot better
   2. A little better
   3. No change
   4. Symptoms got worse
6. Did your symptoms ever return or worsen again after the UAE?
   1. If so, what symptoms returned?
   2. If so, how many years did it take for them to return or worsen after the UAE?
7. Did you have any procedure or surgery to treat fibroid symptoms after the UAE? *(repeat UAE, endometrial ablation, myomectomy, hysterectomy for fibroids)*
   1. If so, what date or approximately what date did this treatment take place?
8. Did you have a hysterectomy that was for something other than fibroids?
   1. If so, what was it for?
   2. If so, what date or approximately what date did this take place?
9. *(Ask question if patient has NOT had a hysterectomy):* Have you entered menopause?
   1. If so, in what year did you have your last period? (Or how old were you?)
   2. If not, are you experiencing perimenopausal symptoms?
10. Did you ever seek to become pregnant after the UAE?
    1. If so, were you successful in having a child?
11. Overall, reflecting on how your fibroid symptoms and quality of life changed after the UAE, how satisfied are you with having a UAE on a scale of 1 to 5? (1= not satisfied at all, 5= very satisfied). Why did you choose that score?
