# Supplementary material for: Long-Term Outcomes of Uterine Artery Embolization for Treatment of Fibroids in Women Under 40: A Retrospective Survey Study at Two Institutions with Median 16-year Follow-up
Source: Cardiovasc Intervent Radiol. 2026 Mar 27;49(7):1391–9. doi: 10.1007/s00270-026-04402-w (PMC13337861; doi:10.1007/s00270-026-04402-w)
Supplement: Supplementary file 2 — Supplementary file2 (DOCX 21 KB) [file 270_2026_4402_MOESM2_ESM.docx]

Appendix B: Kaplan Meier data tables.

Supplemental Table 1. Kaplan Meier data for time to recurrence or worsening of symptoms in patients who reported fibroid symptoms were “a lot” or “a little” better in the first 6 months after UFE.

| Time (years) | Event or Censor |
| --- | --- |
| 0.5 | 1 |
| 0.75 | 1 |
| 1 | 1 |
| 1.5 | 1 |
| 1.75 | 1 |
| 2 | 1 |
| 2 | 1 |
| 2 | 1 |
| 2 | 1 |
| 2 | 1 |
| 2 | 1 |
| 2 | 1 |
| 2.5 | 1 |
| 3 | 1 |
| 3 | 1 |
| 3 | 1 |
| 3 | 1 |
| 3 | 1 |
| 4.5 | 1 |
| 5 | 1 |
| 5 | 1 |
| 5 | 1 |
| 5 | 1 |
| 5.5 | 1 |
| 5.75 | 1 |
| 6 | 1 |
| 6 | 1 |
| 6 | 1 |
| 8 | 1 |
| 8 | 1 |
| 10 | 1 |
| 10 | 1 |
| 10 | 1 |
| 10 | 1 |
| 10 | 1 |
| 11 | 1 |
| 11 | 1 |
| 11 | 1 |
| 12.38 | 0 |
| 12.67 | 0 |
| 12.87 | 0 |
| 13 | 1 |
| 13.12 | 0 |
| 13.30 | 0 |
| 13.56 | 0 |
| 13.98 | 0 |
| 14 | 1 |
| 14.25 | 0 |
| 14.33 | 0 |
| 14.73 | 0 |
| 14.75 | 0 |
| 15.02 | 0 |
| 15.18 | 0 |
| 15.56 | 0 |
| 15.78 | 0 |
| 15.8 | 0 |
| 16.55 | 0 |
| 17.26 | 0 |
| 17.37 | 0 |
| 17.40 | 0 |
| 17.93 | 0 |
| 18.27 | 0 |
| 18.75 | 0 |
| 18.79 | 0 |
| 18.83 | 0 |
| 18.92 | 0 |
| 19.08 | 0 |
| 20 | 1 |
| 20.25 | 0 |
| 20.31 | 0 |
| 22.61 | 0 |

Supplemental Table 2. Kaplan Meier data for time to hysterectomy for recurrence or worsening of symptoms in patients who reported fibroid symptoms were “a lot” or “a little” better in the first 6 months after UFE.

| Time (years) | Event or Censor |
| --- | --- |
| 0.78 | 1 |
| 1.84 | 1 |
| 1.99 | 1 |
| 2.05 | 1 |
| 2.24 | 1 |
| 2.29 | 1 |
| 2.3 | 0 |
| 2.43 | 1 |
| 2.90 | 1 |
| 3.30 | 1 |
| 3.57 | 1 |
| 4.94 | 1 |
| 5.14 | 1 |
| 5.53 | 1 |
| 5.82 | 1 |
| 6.90 | 1 |
| 7.21 | 1 |
| 7.36 | 1 |
| 7.55 | 1 |
| 7.91 | 1 |
| 8.58 | 1 |
| 10.38 | 1 |
| 10.91 | 1 |
| 12.06 | 0 |
| 12.38 | 0 |
| 12.49 | 0 |
| 12.87 | 0 |
| 13.02 | 1 |
| 13.12 | 0 |
| 13.30 | 0 |
| 13.56 | 0 |
| 13.98 | 0 |
| 14.2 | 1 |
| 14.25 | 0 |
| 14.33 | 0 |
| 14.37 | 0 |
| 14.73 | 0 |
| 14.75 | 0 |
| 14.75 | 0 |
| 14.79 | 1 |
| 14.84 | 0 |
| 14.87 | 0 |
| 15.02 | 0 |
| 15.18 | 0 |
| 15.25 | 1 |
| 15.56 | 0 |
| 15.78 | 0 |
| 15.8 | 0 |
| 15.80 | 0 |
| 15.97 | 0 |
| 16.05 | 0 |
| 16.2 | 0 |
| 16.55 | 0 |
| 17.26 | 0 |
| 17.37 | 0 |
| 17.40 | 0 |
| 17.91 | 0 |
| 17.93 | 0 |
| 18.27 | 0 |
| 18.75 | 0 |
| 18.79 | 0 |
| 18.83 | 0 |
| 18.92 | 0 |
| 19.08 | 0 |
| 19.71 | 0 |
| 19.73 | 0 |
| 20.25 | 0 |
| 20.31 | 0 |
| 22.61 | 0 |
| 22.70 | 0 |
| 22.81 | 0 |
